# Multilocus analyses indicate a mosaic distribution of hybrid populations in ground squirrels (genus *Ictidomys*)

**DOI:** 10.1002/ece3.755

**Published:** 2013-10-11

**Authors:** Cody W Thompson, Faisal Ali Anwarali Khan, Frederick B Stangl, Robert J Baker, Robert D Bradley

**Affiliations:** 1 Department of Biological Sciences, Texas Tech UniversityLubbock, Texas, 79409-3131; 2 Natural Science Research Laboratory, Museum of Texas Tech UniversityLubbock, Texas, 79409-3191; 3 Department of Zoology, Faculty of Resource Science and Technology, Universiti Malaysia SarawakKota Samarahan, Sarawak, 94300, Malaysia; 4 Biology Department, Midwestern State UniversityWichita Falls, Texas, 76308

**Keywords:** Amplified fragment length polymorphism, cytochrome-*b*, *Ictidomys*, mosaic hybrid zone, secondary contact, Y-linked structural maintenance of chromosomes

## Abstract

DNA sequence data from mitochondrial cytochrome-*b* (*Cyt*b) and Y-linked structural maintenance of chromosomes (*SmcY*) genes were combined with 478 nuclear loci obtained from amplified fragment length polymorphisms (AFLP) to assess the extent of hybridization and genetic spatial structure of populations in two hybridizing species of ground squirrel (*Ictidomys parvidens* and *Ictidomys tridecemlineatus*). Based on AFLP analyses of 134 individuals from 28 populations, 10 populations were identified that possessed hybrid individuals. Overall estimates of *F*_ST_ values revealed strong support for population structure in the *Cyt*b data set; however, analyses of the *SmcY* gene and the AFLP data indicated ongoing gene flow between species. Pairwise *F*_ST_ comparisons of populations were not significant for the *SmcY* gene; although they were significant for the *Cyt*b gene, indicating that these populations were structured and that gene flow was minimal. Therefore, gene flow between *I. parvidens* and *I. tridecemlineatus* appeared to be restricted to populations that exhibited hybridization. In addition, the fragmented nature of the geographic landscape suggested limited gene flow between populations. As a result, the distributional pattern of interspersed parental and hybrid populations were compatible with a mosaic hybrid zone model. Because ground squirrels display female philopatry and male-biased dispersal, the ecology of these species is compatible with this hypothesis.

## Introduction

Hybrid zones are the result of the interaction of genetically distinct groups that produce offspring of mixed ancestry (Barton and Hewitt 1985; Arntzen 1996; Abbott et al. 2013). Hybrid zones often provide a natural laboratory for addressing major evolutionary concepts (Hewitt 1988; Baker et al. 1989; Harrison 1993). For example, studies of hybrid zones offer the opportunity to examine genetic control of speciation, mechanistic models of maintenance, premating and postmating isolation, direction of introgression, and other processes and patterns of hybridization (Shurtliff 2013). Consequently, understanding the spatial structure of phenotypes and genotypes in a hybrid zone is essential to determine the underlying mechanisms responsible for hybridization (Cain et al. 1999).

The spatial structure of a hybrid zone is classified into two categories: clinal and mosaic (Harrison 1990; Cain et al. 1999). Clinal hybrid zones are defined by a gradual transition of character states from one parental type to the other (Ross and Harrison 2002). Many complex, mechanistic models of maintenance have been described for clinal hybrid zones, including the dynamic equilibrium model (Moore 1977; or tension zone, Barton 1979), ecological gradient (Endler 1977), and bounded hybrid superiority (Moore 1977). Conversely, mosaic hybrid zones are defined by sharp changes in character states as a result of the patchy spatial structure of parental populations and their hybrids. Frequently, mosaic hybrid zones are associated with a heterogeneous environment, generating a patchwork of suitable habitat for hybridizing taxa (Harrison 1986, 1990). Therefore, maintenance of a mosaic hybrid zone includes environmental components (Harrison 1986, 1990); however, the underlying mechanism is not clear (Ross et al. 2008; M'Gonigle and FitzJohn 2010). To understand the processes of hybrid zone maintenance would require a priori knowledge of the spatial structure of the phenotypes and genotypes of hybridizing taxa.

To properly assess the spatial structure of a hybrid zone, an appropriate geographic scale must be determined. Superficially, when examined on a broader geographic scale, a hybrid zone may appear to be widely sympatric and clinal because of the overlapping distribution of species. However, if the same hybrid zone is examined at a narrower geographic scale, the distribution of species may appear parapatric and mosaic in nature (Ross and Harrison 2002). Therefore, hybridizing taxa with limited dispersal capability or that are not highly vagile may appear to be widely sympatric at global or continental scales, but the interspecific interactions required to produce offspring occur at a much smaller spatial scale (Ross and Harrison 2002; Miño et al. 2011). For example, North American field crickets (*Gryllus pennsylvanicus* and *Gryllus firmus*) have been examined on multiple geographic scales (Harrison and Arnold 1982; Harrison 1986; Harrison and Rand 1989; Rand and Harrison 1989; Harrison and Bogdanowicz 1997; Ross and Harrison 2002). At a regional scale, the hybrid zone appeared to be clinal; however, at a smaller geographic scale, the hybrid zone became mosaic in appearance (Ross and Harrison 2002). It is at finer geographic scales that the true mosaic nature of this hybrid zone was most obvious and strongly associated with changes in soil type (Rand and Harrison 1989).

In mammals, little attention has been directed toward understanding this most basic concept of spatial structure; rather, the focus has been on uncovering hybridizing taxa and interpreting the causes for hybridization (Shurtliff 2013). Hybridization between two species of ground squirrel in the genus *Ictidomys* (*Ictidomys parvidens* and *Ictidomys tridecemlineatus*) is a prime example of this type of effect. These species hybridize (Nadler et al. 1971; Zimmerman and Cothran 1976; Cothran et al. 1977; Cothran 1983; Cothran and Honeycutt 1984) and are hypothesized to form a zone of sympatry with localized areas of hybridization across much of the southern Great Plains of southeastern New Mexico and western Texas. However, recent morphologic (Stangl et al. 2012) and genetic (Thompson et al., in press) studies have demonstrated that hybridization was restricted to small, isolated populations along a parapatric boundary. Because much of the southern Great Plains has been modified for row-crop agriculture (Choate 1997), a fragmented patchwork of suitable habitat is available to both species.

Despite evidence for contemporary hybridization, detailed analyses of the mitochondrial cytochrome-*b* (*Cyt*b) gene indicated a common mitochondrial genome with similar haplotypes among individuals of both species across the putative zone of sympatry. Haplotypes of individuals from this region were similar to haplotypes of *I. tridecemlineatus* outside of the putative zone of sympatry (1.91% average between group genetic distance—Thompson et al., in press). Thompson et al. (in press) interpreted this unique mitochondrial genome to be the result of mitochondrial capture due to an ancient hybridization event that occurred ∼0.47 million years ago (mya) during the late Irvingtonian Land Mammal Age, possibly as the result of intense changes in distribution leading to times of sympatry as a result of the climatic oscillations of the Quaternary glacial cycle. Development of modern roadways during the mid-1900s presumably provided the necessary connectivity for dispersal between populations to reestablish hybridizing populations (Cothran 1983). Therefore, contemporary hybridization could be a recent phenomenon and the result of secondary contact (Cothran 1982; Stangl et al. 2012).

Thompson et al. (in press) identified six localities containing hybrid individuals across the putative zone of sympatry through the use of a uniparentally inherited marker, the Y-linked structural maintenance of chromosomes (*SmcY*). The identification of hybrids was confirmed based on the incongruence of haplotypes of the *SmcY* gene and morphological identifications (Stangl et al. 2012; Thompson et al., in press). Nearly, all localities were found within or near human-modified habitats (e.g., cemeteries, golf courses, parks, etc.). Only two localities contained putative hybrids and both parental types (Thompson et al., in press).

Because ground squirrels are limited in their dispersal abilities as a result of reduced activity periods due to extensive hibernation bouts (Inouye et al. 2000), understanding spatial structure of hybridizing populations requires a geographic scale adapted to these constraints. Estimates of dispersal distance among juvenile *I. tridecemlineatus* range from 183 to 267 m from their nest (McCarley 1966). In addition, these two species are colonial, living in groups of approximately 4/ha in the spring and nearly 20/ha following the emergence of offspring in the early summer months (Rongstand 1965; Grant 1972; Mitchell 1972). Therefore, an appropriate geographic scale would be the size of a home range (∼1.42 ha females, ∼4.74 ha males; McCarley 1966), encompassing potential mates and their offspring. The primary objectives of this study were to evaluate the levels of introgression at localities of hybridization (Stangl et al. 2012; Thompson et al., in press) and to determine the spatial structure of hybridizing populations at an appropriate geographic scale. Data previously reported for two uniparentally inherited genes (*Cyt*b and *SmcY*—Thompson et al., in press) and newly obtained data from the biparentally inherited amplified fragment length polymorphisms (AFLP) were used to determine parental types and hybrid classes. AFLPs provide a genome-wide scan and produce a large number of loci for individuals sampled (Bensch and Åkesson 2005), increasing the resolving power of identifying different genotypic classes. By combining the two uniparentally inherited markers with AFLPs, this study addressed the directionality of hybridization and gene flow between hybridizing populations and nearby peripheral populations.

## Materials and Methods

### Sampling

**Figure 1:**
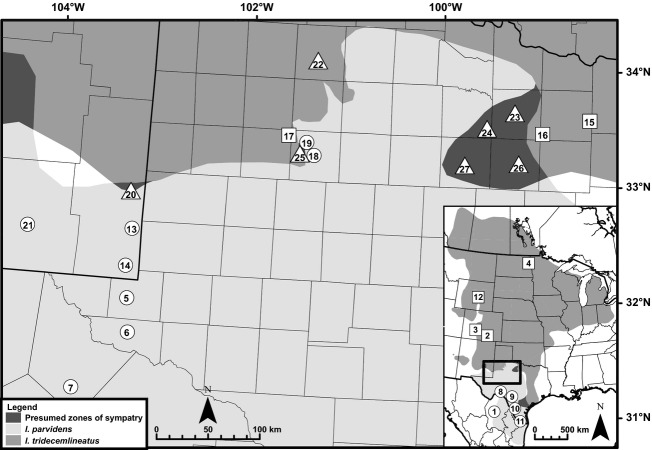
Maps illustrating populations of ground squirrels (genus *Ictidomys*) examined in this study. Maps redrawn from data reported in Thompson et al. (in press). Inset map shows locations of populations across the entire distribution of both species. Large map shows the area in the black box found on the inset map. In both maps, circles indicate locations where all individuals were identified as *Ictidomys parvidens*. Squares indicate locations where all individuals were identified as *Ictidomys tridecemlineatus*. Triangles indicate locations where individuals were morphologically or genetically identified as either species or as hybrids. Numbers within the respective shapes indicates the locality number as referenced in Table 1 and Appendix S1.

Tissue samples were obtained from localities of hybridization as reported in Stangl et al. (2012) and Thompson et al. (in press), as well as from peripheral and reference localities without evidence of known hybridization (see Fig. 1 and Table 1). Peripheral populations were selected based on their close proximity to putative hybrid populations and potential for dispersal between these populations (i.e., habitat suitability and connectivity) or if it was possible to establish a linear transect across the presumed zone of contact to incorporate potential parental types. This sampling strategy resulted in three “pseudotransects” across the presumed zone of contact; however, peripheral populations could not be established for two hybrid populations. In total, 134 tissue samples from 27 localities (Appendix S1) were acquired from Angelo State University Natural History Collections; Midwestern State University; Natural Science Research Laboratory, Museum of Texas Tech University; and New Mexico Museum of Natural History. Three of these samples were from laboratory-reared F_1_ hybrids (Stangl et al. 2012), and together, they were treated as an additional reference locality. All samples were obtained from individuals previously identified morphologically by coat color and pattern and hindfoot length as detailed in Stangl et al. (2012) and implemented by Thompson et al. (in press). These individuals were collected primarily for Stangl et al. (2012) and Thompson et al. (in press); however, samples from some reference populations and a few other localities were from archival tissue collections collected prior to these studies (Stangl et al. 2012; Thompson et al., in press).

**Table 1:** Summary of populations examined based on field, mitochondrial cytochrome-*b* gene (*Cyt*b), Y-linked structural maintenance of chromosomes gene (*SmcY*), and amplified fragment length polymorphisms (AFLP) identifications

Locality #	State	Locality name	Field ID	*Cyt*b ID	*SmcY* ID	AFLP ID
Reference populations						
1	COA	1.5 mi. NW Ocampo	3 par	3 par	3 par	3 par
2	COL	5 mi. S, 5 mi. E Villa Grove	1 tri	1 tri	N/A	1 tri
3	COL	Baca Grande Golf Course	3 tri	3 tri	1 tri	3 tri
4	ND	2 mi. N Arvilla	4 tri	4 tri	2 tri	4 tri
5	TX	Winkler County Airport	1 par	1 par	1 par	1 par
6	TX	Pyote	1 par	1 par	1 par	1 par
7	TX	Saragosa Cemetery	1 par	1 par	N/A	1 par
8	TX	Turkey Track Canyon	1 par	1 par	N/A	1 par
9	TX	1 mi. S Highway 90 on Kingsway in Del Rio	1 par	1 par	N/A	1 par
10	TX	B & M Ranch	1 par	1 par	1 par	1 F_2_
11	TX	Bentsen Rio Grande Valley State Park	1 par	1 par	1 par	1 F_2_
12	WY	Engel King Ranch	1 tri	1 tri	1 tri	1 tri
Peripheral populations						
13	NM	Eunice	2 par	2 tri-like	1 par	2 par
14	NM	Jal	3 par	3 tri-like	2 par	3 par
15	TX	Windthorst	1 tri	1 tri-like	1 tri	1 tri
16	TX	Megargel Cemetery	1 tri	1 tri-like	N/A	1 tri BC
17	TX	3 km S, 0.6 km E Southland	4 tri	4 tri-like	2 tri	4 tri BC
18	TX	Roadside Park	3 par	3 tri-like	1 par	1 par, 1 F_2_, 1 par BC
19	TX	Post	6 par	6 tri-like	2 par	6 par
Putative hybrid populations						
20	NM	Hobbs	29 par, 9 tri	38 tri-like	3 par, 1 tri	25 par, 3 F_1_, 2 F_2_, 8 par BC
21	NM	Carlsbad	11 par	11 par	2 par*	11 par
22	TX	Floydada	5 tri	5 tri-like	1 par, 1 tri	5 tri
23	TX	Seymour	15 hyb, 1 par, 2 tri	18 tri-like	2 par, 1 tri	8 F_1_, 5 F_2_, 3 par BC, 2 tri BC
24	TX	Goree Cemetery	2 hyb, 2 par	4 tri-like	2 par	3 par, 1 par BC
25	TX	Wayne Stewart Ranch	2 par	2 tri-like	1 tri	1 F_1_, 1 tri BC
26	TX	Throckmorton Cemetery	3 hyb, 8 par	11 tri-like	3 par, 1 tri	4 par, 1 F_1_, 1 F_2_, 5 par BC
27	TX	Haskell	1 hyb, 2 par	3 tri-like	N/A	3 par
N/A	N/A	Laboratory F_1_ Hybrids	3 hyb	3 hyb	1 tri	3 F_1_

Locality # corresponds to the numbers depicted in Figure 1 and referenced in Appendix S1. Numbers of specimens identified by each method are indicated for each locality.

Field ID, field identification; *Cyt*b ID, *Cyt*b identification; *SmcY* ID, *SmcY* identification; AFLP ID, AFLP identification; COA, Coahuila; COL, Colorado; ND, North Dakota; NM, New Mexico; TX, Texas; WY, Wyoming; hyb, morphological hybrid; par, *Ictidomys parvidens*; tri, *I. tridecemlineatus*; tri-like, *I. tridecemlineatus*-like *Cyt*b haplotype; F_1_, first filial generation hybrid; F_2_, second filial generation hybrid; par BC, *I. parvidens* backcross hybrid; tri BC, *I. tridecemlineatus* backcross hybrid; and N/A, data not available.

* Thompson et al. (in press) reported an *I. tridecemlineatus SmcY* haplotype at this locality.

### DNA sequences

Sequences for *Cyt*b and *SmcY* genes were obtained from individuals examined in Thompson et al. (in press). These sequences corresponded directly to samples obtained for this study. GenBank accession numbers and corresponding catalog numbers are provided in Appendix S1.

### AFLP genotyping

The AFLP protocol followed techniques in McDonough et al. (2008) and Khan et al. (2010) as modified from Vos et al. (1995). *Ase*I adapter sequences were the same as Khan et al. (2010). A labeled selective *EcoR*I primer was used in combination with seven selective *Ase*I primers as reported in McDonough et al. (2008) to produce unambiguous and distinct AFLPs. Fragments were detected on an ABI 3100-*Avant* and compared with the Genescan-400HD ROX size standard (Applied Biosystems, Foster City, CA). Only fragments (50–400 bp in length) that could be scored unambiguously as present or absent with the program GeneMapper, version 3.7 (Applied Biosystems) were used. The fragments were converted to a binary matrix consisting of zeros and ones using GenAlEx, version 6.5 (Peakall and Smouse 2006). Technical and observer error rates were estimated following the methodology in Bonin et al. (2004). The binary matrix for the AFLP data was deposited in Dryad.

### Data analyses

Statistical parsimony networks were generated from the program TCS 1.21 (Clement et al. 2000) to visualize any population or species-level differentiation. In addition, TCS was used to determine haplotype diversity for the *Cyt*b gene, as well as determine parsimony networks and haplotype diversity for sequences in the *SmcY* data set. Haplotype frequency and nucleotide diversity were estimated for each population for both data sets using the program Arelquin 3.5.1.3 (Excoffier and Lischer 2010). Arlequin also was used to perform an analysis of molecular variance (AMOVA) and to determine population differences by estimating fixation indices (*F*_ST_) for each population and for pairwise comparisons between localities.

A principal coordinate analysis (PCoA) from binary genetic distances and an AMOVA in the program GenAlEx, version 6.5 (Peakall and Smouse 2006) were used to assess patterns of genetic divergence within the AFLP data. The proportion of each individual's admixture (*q*) was estimated with STRUCTURE 2.3.1 (Falush et al. 2007) to determine the extent of introgression between parental genomes (Falush et al. 2003). Therefore, the number of inferred populations (*K*) was set to two to represent both species without imposing any a priori knowledge of species identification (Thompson et al. 2011). The burn-in period and number of iterations in STRUCTURE were set at 10,000 and 50,000, respectively. Five independent runs were performed, and the admixture proportions were averaged.

Although STRUCTURE can be used to estimate hybrid class membership, it is limited in its ability to distinguish between different hybrid classes without imposing arbitrary cutoffs. In addition, it is incapable of identifying multigenerational backcrossed individuals with a history of recent admixture (Randi 2008), which has been suggested for *I. parvidens* and *I. tridecemlineatus* (Thompson et al., in press). Therefore, hybrid class membership was inferred with NewHybrids 1.1 beta (Anderson and Thompson 2002) by estimating the posterior probability of an individual falling into one of 14 hybrid classes (i.e., pure *I. parvidens*, pure *I. tridecemlineatus,* F_1_, F_2_, *I. parvidens* backcross, *I. tridecemlineatus* backcross, *I. parvidens* 2nd-generation backcross, *I. tridecemlineatus* 2nd-generation backcross, *I. parvidens* 3rd-generation backcross, *I. tridecemlineatus* 3rd-generation backcross, *I. parvidens* 4th-generation backcross, *I. tridecemlineatus* 4th-generation backcross, *I. parvidens* 5th-generation backcross, or *I. tridecemlineatus* 5th-generation backcross). Genotype frequencies of each hybrid class were checked for sufficient loci differentiation to ensure reliable hybrid class assignment by the program (Wiley et al. 2009). Test runs were completed initially without a priori knowledge of species identification to ensure reference individuals were categorized as pure parental types. In addition, test runs were completed to determine the effects of using Jeffreys-like or Uniform priors on mixing proportions and allele frequencies. For the complete runs, species identification of reference populations was assigned a priori to estimate allele frequencies for animals of pure origin, and Jeffreys-like priors were used to account for rare alleles (Anderson and Thompson 2002). The number of sweeps for burn-in and after burn-in was set to 10,000 and 50,000, respectively. All other settings were set to the defaults, and five independent runs were averaged to determine likelihood values.

The program AFLP-SURV 1.0 (Vekemans 2002) was used to estimate *F*_ST_ values among and between pairs of localities under the assumption of Hardy–Weinberg equilibrium (HWE). The significance of the *F*_ST_ values was tested against a null distribution determined by 50,000 random permutations of individuals among samples following the method of Lynch and Milligan (1994) and employed by AFLP-SURV (Vekemans 2002). In addition, locality-specific inbreeding coefficients (*F*_IS_) were estimated using the approximate Bayesian computation (ABC) approach in the program ABC4F (Foll et al. 2008). This program relaxes assumptions of HWE, which corrects for the ascertainment biases of AFLP data. The program was parameterized to assume a bias of one for hidden loci and no bias for fixed loci. The sample *F*_IS_ value was determined as the modal value of its posterior distribution. The 95% highest posterior probability density interval (95% HPDI) of the *F*_IS_ distribution was used to detect any departure from HWE with a threshold value of 0.001 for acceptance (Gagnaire et al. 2011). Departure from HWE was verified by testing for linkage disequilibrium with the program LIAN 3.1 (Haubold and Hudson 2000). LIAN uses the standardized index of association (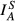) to provide a genome-wide measure of multilocus linkage disequilibrium, which compares the variance of the distribution of pairwise mismatch values with the null distribution of the variance under linkage equilibrium. The comparison of these distributions was completed through a Monte Carlo simulation test 10,000 iterations of resampling. Comparisons were made for the entire data set and for each population with a suitable sample size.

## Results

### Sequence diversity and network analysis

The parsimony network analysis of the *Cyt*b gene identified 79 haplotypes within the 134 sequences of the data set (Appendix S1) and resulted in two unconnected networks (not shown). The first network consisted of individuals identified morphologically as *I. parvidens*. This network was composed primarily of individuals from reference populations. The second network contained individuals identified morphologically as *I. parvidens*, *I. tridecemlineatus*, and hybrids (Thompson et al., in press). These individuals were from populations with potential hybrids, as well as reference populations of *I. tridecemlineatus*. The number of haplotypes present at each locality was greatest in populations with the highest sample sizes (Table 2); however, nucleotide diversity (π) was low for all localities (0.0007–0.0097; Table 2). Results of the AMOVA for the *Cyt*b data set indicated significant variation among and between populations (*P* < 0.05). The genetic variation among populations was 78.56%, whereas genetic variation within populations was 21.44%.

**Table 2:** Summary of the statistics from population analyses for localities examined. Locality number and name corresponds to those found in Table 1 and shown in Figure 1

			*Cyt*b		*SmcY*		AFLP		
					
Locality #	Locality Name	*N*	# Haplotypes	*π*	# Haplotypes	*π*	*H*_e_	*F*_IS_	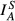
1	1.5 mi. NW Ocampo	3	3	0.0023	1	0	0.38638	0.12*	−0.0020
2	5 mi. S, 5 mi. E Villa Grove	1	1	0	N/A	N/A	0.52939	N/A	N/A
3	Baca Grande Golf Course	3	3	0.0018	1	0	0.39451	0.095*	−0.0007
4	2 mi. N Arvilla	4	2	0.0009	2	0.0027	0.35181	0.865*	0.0366**
5	Winkler County Airport	1	1	0	1	0	0.53313	N/A	N/A
6	Pyote	1	1	0	1	0	0.53227	N/A	N/A
7	Saragosa Cemetery	1	1	0	N/A	N/A	0.53083	N/A	N/A
8	Turkey Track Canyon	1	1	0	N/A	N/A	0.53313	N/A	N/A
9	1 mi. S Highway 90 on Kingsway in Del Rio	1	1	0	N/A	N/A	0.53342	N/A	N/A
10	B & M Ranch	1	1	0	1	0	0.52795	N/A	N/A
11	Bentsen Rio Grande Valley State Park	1	1	0	1	0	0.52594	N/A	N/A
12	Engel King Ranch	1	1	0	1	0	0.52623	N/A	N/A
13	Eunice	2	2	0.0053	1	0	0.44290	0.11*	N/A
14	Jal	3	3	0.0018	1	0	0.39620	0.0851*	0.0008
15	Windthorst	1	1	0	1	0	0.53025	N/A	N/A
16	Megargel Cemetery	1	1	0	N/A	N/A	0.52968	N/A	N/A
17	3 km S, 0.6 km E Southland	4	3	0.0023	2	0.0027	0.37146	0.1*	0.0069**
18	Roadside Park	3	3	0.0018	1	0	0.39411	0.095*	0.0199**
19	Post	6	6	0.0026	1	0	0.31383	0.0851*	0.0052**
20	Hobbs	38	24	0.0058	3	0.0412	0.19755	0.075*	0.0065**
21	Carlsbad	11	8	0.0016	1	0	0.24099	0.13*	0.0186**
22	Floydada	5	3	0.0011	3	0.0901	0.34535	0.0997*	0.0039**
23	Seymour	18	10	0.0030	3	0.0528	0.25716	0.12*	0.0034**
24	Goree Cemetery	4	3	0.0007	1	0	0.36165	0.105*	0.0056
25	Wayne Stewart Ranch	2	2	0.0097	N/A	N/A	0.46623	N/A	N/A
26	Throckmorton Cemetery	11	5	0.0028	4	0.0451	0.26874	0.0851*	0.0077**
27	Haskell	3	2	0.0030	N/A	N/A	0.39439	0.085*	N/A
N/A	Laboratory F_1_ Hybrids	3	1	0	1	0	0.40211	0.23*	0.0002

Population statistics included are haplotype frequency, nucleotide diversity (*π*), expected heterozygosity (*H*_e_), inbreeding coefficient (*F*_IS_), and multilocus linkage disequilibrium (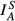).

*Cyt*b, mitochondrial cytochrome-*b* gene; *SmcY*, Y-linked structural maintenance of chromosomes gene; AFLP, amplified fragment length polymorphism; *N*, sample size; *π*, nucleotide diversity; *H*_e_, expected heterozygosity; *F*_IS_, inbreeding coefficient;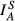, multilocus linkage disequilibrium; and N/A, data not available.

* Hardy-Weinberg equilibrium accepted, **significant levels of linkage disequilibrium.

The parsimony network analysis of the 39 sequences of the *SmcY* data set (Appendix S1) identified 17 haplotypes. This analysis resulted in three unconnected networks (not shown). The first network was comprised primarily of individuals identified morphologically as *I. parvidens*. The second network consisted primarily of individuals identified morphologically as *I. tridecemlineatus*. Both networks contained a few individuals representative of the other taxon, as well as putative morphologic and genetic hybrids. The third network contained individuals from a reference population of *I. tridecemlineatus* in North Dakota. Generally, each locality was represented by a single haplotype, and no locality had more than four haplotypes. Because of the low numbers of sequences and subsequent haplotypes, nucleotide diversity could be determined only for six of the 28 localities. Four of these localities had high nucleotide diversity (0.0027–0.0901), as a result of the presence of *SmcY* haplotypes of both species (Table 2). The results of the AMOVA indicated significant variation among and between populations (*P* < 0.05). The variation within populations accounted for 65.04% of the total variation, and the variation among populations accounted for 34.96% of the total variation.

### Patterns of AFLP variation

A total of 478 AFLP fragments were scored, and 300 fragments were polymorphic (62.2%). Of the polymorphic fragments, 10 fragments were determined to be fixed through species-level comparisons of reference populations and diagnostic in distinguishing each species. The majority of these markers (8 of 10) were present in reference populations of *I. parvidens* but were absent in reference populations of *I. tridecemlineatus*.

**Figure 2:**
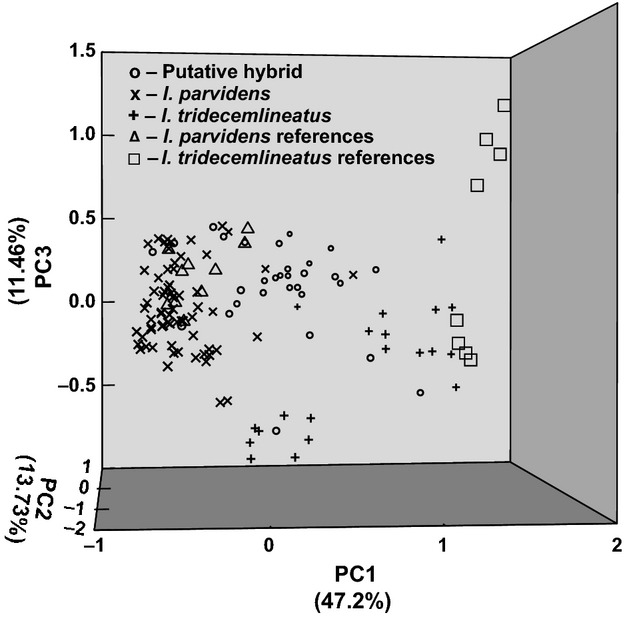
Plot of the first three coordinates of the principal coordinate analysis based on amplified fragment length polymorphisms (AFLP) data. The first three principal coordinates explained 72.4% of the cumulative variation in the AFLP data set. PC1 accounted for 47.2% of the variation, whereas PC2 and PC3 accounted for 13.73% and 11.46% of the variation, respectively.

The individuals examined for the PCoA analysis were placed in one of three groups (*I. parvidens*, *I. tridecemlineatus*, or hybrid) as designated in Thompson et al. (in press), resulting in three overlapping clusters that represented each group (Fig. 2). Reference populations of both species clustered within their respective groups. The majority of the putative hybrids (as determined by Thompson et al., in press) grouped intermediate to the clusters containing reference individuals. The first three axes of the PCoA explained 72.4% of the cumulative variation in the AFLP data set. The first axis accounted for 47.2% of the variation, whereas the second and third axes accounted for 13.73% and 11.46%, respectively.

Because of small sample size at some localities, the AMOVA was performed treating each species and hybrids as populations as in the PCoA. This approach revealed significant genetic variation among and between populations (*P* = 0.001). The percentage of molecular variation among populations was 19%, whereas the percentage of molecular variation within populations was 81%. The majority of the molecular variation occurred between the *I. parvidens* group and *I. tridecemlineatus* group (ϕ_PT_ = 0.275, *P* = 0.001). The molecular variation between the hybrids group and the *I. parvidens* (ϕ_PT_ = 0.130, *P* = 0.001) and *I. tridecemlineatus* (ϕ_PT_ = 0.111, *P* = 0.001) groups was lower.

### Extent of hybridization and hybrid class membership

**Figure 3:**
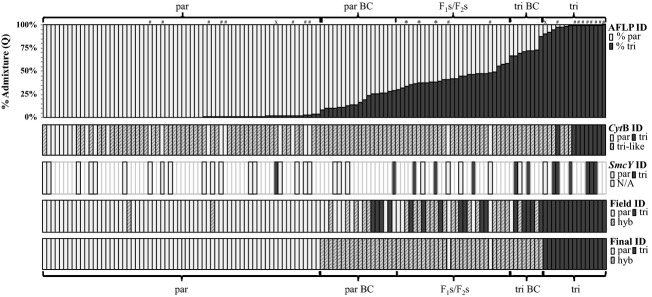
Identification of individuals examined based on field, mitochondrial cytochrome-*b* gene (*Cyt*b), Y-linked structural maintenance of chromosomes gene (*SmcY*), and amplified fragment length polymorphisms (AFLP). Field, *Cyt*b, and *SmcY* identifications follow those reported in Thompson et al. (in press). AFLP data are represented in a histogram by admixture values (Q; percentage of parental genome). Parental or hybrid identification for the AFLP data as determined by the program NewHybrids 1.1 beta (Anderson and Thompson 2002) is shown above the histogram. par, *Ictidomys parvidens*; par BC, *I. parvidens* backcross; F_1_, F_1_ hybrid; F_2_, F_2_ hybrid; tri BC, *Ictidomys tridecemlineatus* backcross; tri, *I. tridecemlineatus*; *, laboratory-reared F_1_ hybrid;% par, percentage of *I. parvidens* genome;% tri, percentage of *I. tridecemlineatus* genome; tri-like, *I. tridecemlineatus*-like *Cyt*b haplotype; N/A, data unavailable; hyb, hybrid; X, multigenerational backcross; #, reference individual.

Admixture values (*q*) estimated from the program STRUCTURE indicated that 73.2% of individuals sampled clustered with *I. parvidens*, whereas 26.8% of individuals sampled clustered with *I. tridecemlineatus*. However, values of *q* ranged from 0.0–0.1 (*I. parvidens*) to 0.9–1.0 (*I. tridecemlineatus*) for 81 individuals, likely representing nonadmixed individuals (Vähä and Primmer 2006; see Fig. 3). Most individuals from reference populations (excluding individuals from localities 10 and 11) grouped within these thresholds. Fifty-three individuals (39.55%) possessed values of *q* from 0.1 to 0.9, indicative of admixed individuals (Gompert et al. 2010). The majority of admixed individuals were found within the localities possessing putative hybrids (Stangl et al. 2012; Thompson et al., in press).

The NewHybrids analysis provided further resolution in identification of admixed individuals. Fifty-two of the 134 individuals (17 F_1_s, 11 F_2_s, 16 *I. parvidens* backcrosses, and eight *I. tridecemlineatus* backcrosses) analyzed were identified as hybrids with NewHybrids. No individuals were assigned as multigenerational backcrosses. When considering along with the admixture values from STRUCTURE, 53 of the 134 individuals (16 F_1_s, 11 F_2_s, 18 *I. parvidens* backcrosses, and eight *I. tridecemlineatus* backcrosses) were classified as hybrids based on the AFLP data (Table 1). These individuals were found at 10 localities (10, 11, 16, 17, 18, 20, 23, 24, 25, and 26). Two of these localities (10 and 11) were in southern Texas and were considered to be reference populations. In addition, three localities (21, 22, and 27) with previous reports (Stangl et al. 2012; Thompson et al., in press) of hybrid individuals did not contain any individuals identified as hybrids (see Fig. 1).

When considering the *Cyt*b and *SmcY* haplotypes for individuals, as well as including the field identifications for the specimens examined, the assignment of parental types and hybrid class membership remained essentially the same (Fig. 3). However, two additional individuals (TTU115524 and TTU115600) represented multigenerational backcross hybrids. Although analyses of the AFLP data indicate they are pure parental types (TTU115524 = *I. parvidens*, TTU115600 = *I. tridecemlineatus*), these two individuals have *SmcY* haplotypes incongruent with their nuclear genome, and they are from localities (20 and 23) documented for potential hybridization (Thompson et al., in press). The two individuals from reference localities 10 and 11 (ASNHC11371 and ASNHC13686), however, probably are not hybrids, as they are ∼300 km from the nearest area of presumed sympatry and ∼600 km from the nearest area of known hybridization. Their genotypes likely are due to the retention of ancestral polymorphisms. Therefore, these latter two individuals were not considered to be hybrids in this study.

### Analyses of population differentiation

Overall, estimates of *F*_ST_ values for the *Cyt*b data set were high, indicating considerable population differentiation. The fixation index for the total data set was 0.78562 (*P* < 0.05). In general, pairwise *F*_ST_ estimates were high (−0.57143 to 1.0000), and many pairwise comparisons were statistically significant (86 of 375). In contrast, the estimates of *F*_ST_ values for the *SmcY* data set were lower than those for the *Cyt*b data set, suggesting a high level of outbreeding. The overall *F*_ST_ estimate was 0.03519 (*P* < 0.05). Although pairwise comparisons varied from −1.00000 to 1.00000, only two of the 210 comparisons were statistically significant, indicating a paucity of haplotypes at each locality.

The overall estimated *F*_ST_ value for the AFLP data set was low (*F*_ST_ = −0.0632), indicating a higher than expected number of heterozygotes because of outbreeding. In contrast, the permutation test implemented in the program AFLP-SURV (Vekemans 2002) rejected the null hypothesis of no genetic differentiation among populations (*P* < 0.05), contradicting the result of the overall estimate. However, the pairwise *F*_ST_ comparisons between populations were low (0.0051–0.1988) and statistically significant reflecting a high level of interbreeding between populations, confirming the results of the overall *F*_ST_ estimate for the AFLP data set.

Reliable *F*_IS_ values were estimated for 15 of the 28 populations (Table 2). Estimated *F*_IS_ values for populations ranged from a mode of 0.075 (Locality 20) to 0.865 (Locality 4). All reliable *F*_IS_ values had 95% HDPI that incorporated the 0.001 threshold; therefore, HWE was accepted. Tests for linkage disequilibrium confirmed this result. An analysis of the entire AFLP data set yielded a statistically significant probability of linkage disequilibrium (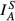 = 0.0186; *P* < 0.00001), supporting the acceptance of HWE by the *F*_IS_ estimates. Estimates of linkage disequilibrium were calculated for 14 localities (Table 2). However, significant levels of linkage disequilibrium only were detected for nine populations. The remaining 14 localities had sample sizes too small to estimate linkage disequilibria.

## Discussion

This study provides strong evidence for contemporary hybridization between *I. parvidens* and *I. tridecemlineatus* in southeastern New Mexico and western Texas. Although it has been hypothesized that these two species have experienced an ancient hybridization event approximately 0.47 mya (Thompson et al., in press), these data suggest that this timeframe was not adequate to allow for complete genetic isolation. Rather, the extent of admixture of the nuclear genome as indicated by the AFLP data suggests that pre- and postmating isolating mechanisms have not developed fully, allowing for the continued formation of hybrids as a result of secondary contact. Cothran (1982) and Stangl et al. (2012) suggested that secondary contact was recent, possibly as a result of the modification of habitat in southeastern New Mexico and western Texas. In addition, the development of roadways provided possible corridors for migration between populations that were isolated as a result of habitat constraints (Cothran 1982). Given that the majority of samples examined in this study were collected from modified environments found in cities and towns (e.g., cemeteries, golf courses, parks, etc.), anthropogenic effects on the landscape may have impacted their ability to hybridize. However, these situations are limited because of the vastness of the unsuitable habitat for either species as a result of modifications for row-crop agriculture in this region (Choate 1997). Therefore, a patchwork of suitable habitat (and opportunities) exists for both species to come into contact and hybridize.

Further evidence supporting contemporary hybridization was evident in the analyses of population structure. Estimation of *F*_ST_ values for the *Cyt*b data set indicated a high level of population differentiation overall and between populations. Because ground squirrels exhibit female philopatry (Mandier and Gouat 1996; Ermakov et al. 2002, 2006), this result is congruent with the ecology of these two species. The overall patchy nature of populations (Stangl et al. 2012) would limit the level of gene flow between those populations with active hybridization and peripheral populations located adjacently (Patton and Smith 1994; Jones et al. 1995), thereby isolating hybridization geographically in a patchy framework. However, ground squirrels are known male-biased dispersers (Holekamp and Sherman 1989; Devillard et al. 2004); therefore, gene flow between populations would be as a result of juvenile male dispersal into adjacent populations. The estimation of the overall *F*_ST_ value for the *SmcY* data set is compatible with this hypothesis, as little population differentiation was detected. Although this result strongly supports gene flow between populations, it does not take into account the lack of introgression of *SmcY* haplotypes at populations with active hybridization.

Thompson et al. (in press) showed that variation was low among haplotypes of the *SmcY* gene in both species, biasing results toward lower *F*_ST_ values. In addition, Thompson et al. (in press) documented the presence of species-specific indels among haplotypes, enabling the determination of introgressing populations. When considering this information, it cannot be confirmed that male-biased dispersal has had a major impact on gene flow between the populations examined; however, the presence of bidirectional hybridization can be determined given that species-specific haplotypes of the *SmcY* gene are present in several hybridizing populations and in apparent multigenerational backcrosses. Therefore, limited gene flow must be occurring to allow for the formation of hybrid individuals.

The analyses of the AFLP data confirm gene flow between populations of *I. parvidens* and *I. tridecemlineatus*. This is evident from the large percentage of admixed individuals (39.55%; Fig. 3) found in the STRUCTURE analysis (Falush et al. 2007). The majority of these individuals were identified unequivocally as hybrids by the NewHybrids program (Anderson and Thompson 2002). In addition, despite contradicting results of the *F*_ST_ estimates, analyses of population structure indicate that structure is minimal, suggesting high levels of migration and subsequent gene flow between populations. These contradicting results are either the product of low and/or unequal sample sizes among populations (see Table 1), thereby producing the negative *F*_ST_ estimate. The calculations of expected heterozygosity (*H*_e_; Table 2) used to estimate the *F*_ST_ values confirmed this hypothesis, as *H*_e_ values for populations with low sample sizes are much higher than those with large sample sizes. Therefore, the effect of the *F*_ST_ values for between population estimates outweighed the *F*_ST_ values for among population estimates leading to the discrepancy in the results. Conversely, the results from the *F*_IS_ estimates and linkage disequilibrium tests support HWE and the nonrandom association of alleles, suggesting that gene exchange at the population level is negligible. Furthermore, the species-level analyses of variance (i.e., PCoA and AMOVA) indicated that *I. parvidens* and *I. tridecemlineatus* have unique genomes; therefore, any gene flow is limited to a few populations. This pattern is aligned closely to the distribution of *SmcY* haplotypes examined in this study and that of Thompson et al. (in press), supporting the hypothesis that males are the primary source of gene flow between the two species.

When considered independently, the results of the population structure analyses for each data set provide contradictory hypotheses to the extent of structure and gene flow. However, when considered together, these patterns offer a clear representation of the spatial structure of hybridizing populations of *I. parvidens* and *I. tridecemlineatus*. Therefore, it appears that ground squirrel populations in the study area are structured genetically. The *Cyt*b data set supports this hypothesis, which might be exaggerated due to the maternal inheritance of the mitochondrial genome and the intense female philopatry in ground squirrels (Mandier and Gouat 1996; Ermakov et al. 2002, 2006). However, the overall patchiness for the habitat in the study area (Choate 1997) would contribute to the distinction in populations and support results of the *Cyt*b analyses. The lack of structure in the *SmcY* and AFLP data sets appear to be directly associated to the presence of populations with active hybridization, and the lack of significance in the pairwise comparisons for *F*_ST_ estimates in the *SmcY* data set suggests that gene flow is limited beyond situations where hybridization occurs. Although this produces a situation for the exchange of genetic material and allows for intense introgression of the nuclear genome, this does not transcend beyond distinct locations of contact. Therefore, any presence of a hybrid zone would have a mosaic spatial structure.

Analyzing this system at a geographic scale relative to the biology of these two species has allowed for a proper assessment of its spatial structure. For example, individuals in locality 23 (*N* = 18) all were classified as hybrids. In fact, all major hybrid classes (i.e., F_1_, F_2_, *I. parvidens* backcross, and *I. tridecemlineatus* backcross) are represented at this locality. Although this may be indicative of a hybrid swarm (Runck et al. 2009), the spatial structure of this hybrid population relative to other hybrid localities was patchy and mosaic in nature (Harrison and Rand 1989). Populations adjacent to locality 23 (localities 15, 16, 24, 26, and 27) either have a few hybrids (mainly backcrossed individuals) or no hybrids at all (Table 1). Similar results occur between locality 25 and nearby populations that border the edge of the Llano Estacado. Thompson et al. (in press) suggested that this physiogeographic boundary might be acting as a boundary between these two species. Because of the orientation of populations with hybrids (localities 17, 19, and 25) to those without (locality 18), it is possible that this natural boundary is serving as a barrier between *I. parvidens* and *I. tridecemlineatus*, especially given the lack of agricultural development near the edge of the Llano Estacado (Choate 1997). However, these populations were quite distant from each other and showed no significant *F*_ST_ estimates of the *SmcY* gene, so gene flow between these populations was minimal.

Locality 20 offers the best example of the mosaic spatial structure. This locality is within the city limits of Hobbs, New Mexico. Although hybrids were found at this locality, they were segregated into several allopatric populations located in green space (cemeteries, golf courses, parks, etc.) throughout the city. The majority of hybrids at this locality were collected at a golf course near the northern edge of the city; however, remaining populations were nearly all *I. parvidens*. Therefore, gene flow likely was limited because of the inability for regular dispersal between populations as a result of the extreme heterogeneous situation of an urban environment. All of these examples support the mosaic designation of this hybrid zone. However, at a spatial scale not conducive to the biology of these two species, these locations would have been considered part of a clinal transition and would have been improperly designated as such.

This study provides the groundwork for future studies of evolutionary mechanisms underlying the speciation process in ground squirrels. Although contemporary hybridization occurs between *I. parvidens* and *I. tridecemlineatus*, the limited geographic extent of hybridizing populations provides an interesting caveat to the stage of the speciation process. Without the addition of the AFLP data set to the uniparentally inherited sequence data, a complete interpretation of the parental contribution to contemporary hybridization could not be inferred, nor could an accurate assessment of spatial structure of parental populations and their hybrids be determined. Because contemporary hybridization is likely the result of secondary contact (Cothran 1982; Stangl et al. 2012), the current mosaic spatial structure of hybridizing populations possibly resulted from a period of isolation with differing selection pressures acting on each distinct population (Abbott et al. 2013). The ancient hybridization hypothesis suggested by Thompson et al. (in press) supports this assumption. Consequently, if these species remained in isolation until recently (Cothran 1982), selection pressures might not have contributed to the evolution of reproductive-isolating mechanisms (Dobzhansky 1940), allowing for continued interspecific reproduction and gene exchange (Baker and Bradley 2006). However, lack of reproductive isolation does not imply genetic isolation (Baker and Bradley 2006), as the nuclear genome of these two species was distinct in nonhybridizing populations. Therefore, understanding the genetic mechanisms underlying hybridization between species would require a thorough examination of reproductive traits between hybridizing populations and adjacent populations (Abbott et al. 2013). With the availability of the *I. tridecemlineatus* genome (Flicek et al. 2008), this system offers an opportunity to do such in the future.

## Data accessibility

Sequence data are available through GenBank. GenBank accession numbers are provided in Appendix S1. AFLP data are deposited in Dryad: doi:10.5061/dryad.g195d.

## Supporting Information

**Appendix S1.** List of specimens examined in this study.
