# Supplementary material for: Multilocus analyses indicate a mosaic distribution of hybrid populations in ground squirrels (genus Ictidomys)
Source: Ecol Evol. 2013 Oct 11;3(13):4448–60. doi: 10.1002/ece3.755 (PMC3856745; doi:10.1002/ece3.755)
Supplement: Supplementary file 1 [file ece30003-4448-SD1.doc]

**Appendix SI** List of specimens examined in this study. Specimens are arranged based on identifications as determined in this study. DNA sequences for the mitochondrial cytochrome-*b* (*Cyt*b) gene and the Y-linked structural maintenance of chromosomes (*SmcY*) were obtained from a previously published study via GenBank (Thompson et al., in press). Specimens are organized by scientific name and by locality number (Fig. 1 and Table 1). Individual specimen numbers are separated by a forward slash (/) and ordered as follows: museum catalog number/*Cyt*b GenBank number/*SmcY* GenBank number. Commas (,) separate specimen numbers of localities with multiple individuals. Specimens without *Cyt*b or *SmcY* sequences are noted with an em dash (—). Museum catalog numbers followed by a letter (e.g., TTU115510A) represent an embryo of the corresponding specimen (e.g., embryo A of TTU155510). Museum collection acronyms used are as follows: ASNHC = Angelo State University, Natural History Collection; MWSU = Midwestern State University; NMMNH = New Mexico Museum of Natural History; and TTU = Natural Science Research Laboratory, Museum of Texas Tech University.

*Ictidomys parvidens*

MEXICO.—(Locality 1) Coahuila: Ocampo Municipality; 1.5 mi. NW Ocampo (NMMNH3679/JX902675/JX903039, NMMNH3680/JX902676/JX903040, NMMNH3715/JX902677/JX903041).

UNITED STATES.—(Locality 20) New Mexico: Lea Co.; Hobbs (TTU54338/JX902688/—); Hobbs, Bensing Park (TTU115515/JX902744/JX903052, TTU115516/JX902745/—); Hobbs, Prairie Haven Cemetery (TTU115512/JX902723/—, TTU115512A/JX902724/—, TTU115512B/JX902725/—, TTU115512C/JX902726/—, TTU115512D/JX902727/—, TTU115512E/JX902728/—, TTU115513/JX902729/—, TTU115513A/JX902730/—, TTU115513B/JX902731/—, TTU115513C/JX902732/—, TTU115513D/JX902733/—, TTU115513E/JX902734/—, TTU115513F/JX902735/—, TTU115513G/JX902736/—, TTU115514/JX902737/—, TTU115514A/JX902738/—, TTU115514B/JX902739/—, TTU115514C/JX902740/—, TTU115514D/JX902741/—, TTU115514E/JX902742/—, TTU115514F/JX902743/—, TTU115523/JX902751/—). (Locality 13) New Mexico: Lea Co.; Eunice, Municipal Park (TTU115521/JX902749/JX903055, TTU115522/JX902750/—). (Locality 21) New Mexico: Eddy Co.; Carlsbad (MWSU22378/JX902632/—, MWSU22379/JX902633/JX903020); Carlsbad, Pecos River Park (TTU115509/JX902714/JX903050, TTU115510/JX902715/—, TTU115510B/JX902716/—, TTU115510C/JX902717/—, TTU115510D/JX902718/—, TTU115510E/JX902719/—, TTU115510F/JX902720/—, TTU115510G/JX902721/—, TTU115511/JX902722/JX903051). (Locality 14) New Mexico: Lea Co.; Jal, City Park (TTU115518/JX902746/JX903053, TTU115519/JX902747/—, TTU115520/JX902748/JX903054). (Locality 24) Texas: Knox Co.; Goree Cemetery (MWSU22369/JX902625/—, MWSU22370/JX902626/—). (Locality 18) Texas: Garza Co.; 2.5 km N, 5 km W Post, Roadside Park (TTU115552/JX902780/JX903063, TTU115553/JX902781/—, TTU115555/JX902783/—). (Locality 19) Texas: Garza Co.; Post, Nichols Park (TTU115550/JX902778/—, TTU115551/JX902779/—); Post Cemetery (MWSU22419/JX902650/—, MWSU22420/JX902651/—, MWSU22423/JX902654/JX903030, MWSU22424/JX902655/JX903031). (Locality 26) Texas: Throckmorton Co.; Throckmorton Cemetery (MWSU22291/JX902595/—, MWSU22357/JX902615/JX903012, MWSU22358/JX902616/JX903013, MWSU22362/JX902619/—). (Locality 27) Texas: Haskell Co.; Haskell (MWSU22282/JX902593/—, MWSU22283/JX902594/—). (Locality 5) Texas: Winkler Co.; 0.5 mi. NW Winkler County Airport (TTU40683/JX902682/JX903043). (Locality 6) Texas: Ward Co.; Pyote, City Park (TTU115580/JX902797/JX903068). (Locality 7) Texas: Reeves Co.; 0.6 km N, 0.25 km E Saragosa, Saragosa Cemetery (TTU115576/JX902793/—). (Locality 8) Texas: Val Verde Co.; 0.5 mi. W Devil's River State Natural Area, Turkey Track Canyon (ASNHC10697/JX902589/—). (Locality 9) Texas: Val Verde Co.; 1 mi. S Hwy 90 on Kingsway in Del Rio (TTU83634/JX902710/—). (Locality 10) Texas: Dimmit Co.; 13 mi. W Artesia Wells, B & M Ranch (ASNHC11371/JX902591/JX903001). (Locality 11) Texas: Hidalgo Co.; Bentsen Rio Grande Valley State Park (ASNHC13686/JX902592/JX903002). (Locality 24) Texas: Knox Co.; Goree Cemetery (MWSU22372/KC011082 /KC011088). (Locality 27) Texas: Haskell Co.; Haskell (MWSU22284/JX902974/—).

*Ictidomys tridecemlineatus*

UNITED STATES.—(Locality 2) Colorado: Saguache Co.; 5 mi. S, 5 mi. E Villa Grove (TTU54368/JX902880/—). (Locality 3) Colorado: Saguache Co.; 0.6 mi. N, 3.5 mi. E Crestone, Baca Grande Golf Course (TTU54369/JX902881/JX903095, TTU54370/JX902882/—, TTU54372/JX902884/—). (Locality 4) North Dakota: Grand Forks Co.; 2 mi. N Arvilla (ASNHC9109/JX902804/JX903069, ASNHC9110/JX902805/JX903070, ASNHC9111/JX902806/—, ASNHC9148/JX902807/—). (Locality 12) Wyoming: Natrona Co.; 6.5 mi. W Casper, Engel King Ranch (ASNHC15066/JX902809/JX903072). (Locality 15) Texas: Archer Co.; Windthorst (MWSU22443/JX902852/JX903086). (Locality 22) Texas: Floyd Co.; Floydada, Floydada Park (TTU115601/JX902949/—); Floydada Cemetery (MWSU22429/JX902843/—, MWSU22430/JX902844/JX903084, MWSU22431/JX902845/—).

*Ictidomys parvidens* x *Ictidomys tridecemlineatus* hybrids

UNITED STATES.—(Locality 16) Texas: Archer Co.; Megargel Cemetery (MWSU22441/JX902851/—). (Locality 17) Texas: Garza Co.; 3 km S, 0.6 km E Southland (TTU115604/JX902952/JX903107, TTU115605/JX902953/—, TTU115606/KC011081/JX903108, TTU115608/JX902955/—). (Locality 18) Texas: Garza Co.; 2.5 km N, 5 km W Post, Roadside Park (TTU115553/JX902781/—, TTU115555/JX902783/—). (Locality 20) New Mexico: Lea Co.; Hobbs (TTU35812/JX902678/—); Hobbs, Ocotilla Park Golf Course (TTU115517/JX902929/JX903105, TTU115524/JX902752/KC011089, TTU115525/JX902753/—, TTU115587/JX902930/—, TTU115587A/JX902931/—, TTU115587B/JX902932/—, TTU115587C/JX902933/—, TTU115587D/JX902934/—, TTU115587E/JX902935/—, TTU115587F/JX902936/—, TTU115587G/JX902937/—, TTU115588/JX902996/JX903120). (Locality 22) Texas: Floyd Co.; Floydada, Floydada Park (TTU115600/JX902997/JX903121). (Locality 23) Texas: Baylor Co.; 2.5 km S, 1.25 km E Seymour, Masonic Cemetery (TTU115593/JX902942/—, TTU115595/JX902944/—); 2.5 km S, 1.25 km E Seymour, Woodmen Cemetery (TTU115592/JX902941/KC011090); Seymour, Catholic Cemetery (MWSU22440/JX902992/—); Seymour, Masonic Cemetery (MWSU22327/JX902596/—, MWSU22328/JX902978/—, MWSU22329/JX902979/—, MWSU22330/JX902980/—, MWSU22331/JX902981/—, MWSU22332/JX902982/—, MWSU22333/JX902983/—, MWSU22334/JX902984/—, MWSU22335/JX902985/—, MWSU22413/JX902990/—, MWSU22414/JX902991/—); Seymour, Old Seymour Cemetery (MWSU22412/JX902989/—); Seymour, Salt Creek Golf Course (MWSU22788/JX902994/JX903118, MWSU22789/JX902995/JX903119). (Locality 24) Texas: Knox Co.; Goree Cemetery (MWSU22371/JX902987/JX903115). (Locality 25) Texas: Garza Co.; 4 mi. W Post, Wayne Stewart Ranch (TTU54339/JX902689/—, TTU54340/JX902993/JX903117). (Locality 26) Texas: Throckmorton Co.; Throckmorton Cemetery (MWSU22285/JX902975/JX903114, MWSU22286/JX902976/—, MWSU22287/JX902977/—, MWSU22359/JX902617/—, MWSU22360/JX902986/—, MWSU22361/JX902618/—). (Laboratory Bred) Texas (MWSU22564/JX902998/—, MWSU22565/JX902999/—, MWSU22566/JX903000/JX903122; MWSU22793/JX902674/JX903038).
